# Save the children by treating their mothers (PriVileG-M-study) - study protocol: a sequentially randomized controlled trial of individualized psychotherapy and telemedicine to reduce mental stress in pregnant women and young mothers and to improve Child’s health

**DOI:** 10.1186/s12888-019-2279-0

**Published:** 2019-11-27

**Authors:** M. Bischoff, V. Howland, J. Klinger-König, S. Tomczyk, S. Schmidt, M. Zygmunt, M. Heckmann, N. van den Berg, B. Bethke, J. Corleis, S. Günther, K. Liutkus, U. Stentzel, A. Neumann, P. Penndorf, T. Ludwig, E. Hammer, T. Winter, H. J. Grabe

**Affiliations:** 1 Department of Health and Prevention, Institute of Psychology, University Greifswald, Greifswald, Germany; 2 Department of Neonatology and Paediatric Intensive Care, University Medicine Greifswald, Greifswald, Germany; 3 Department of Psychiatry and Psychotherapy, University Medicine Greifswald, Greifswald, Germany; 4 Clinic and Polyclinic for Obstetrics and Gynecology, University Medicine Greifswald, Greifswald, Germany; 5 Institute for Community Medicine, University Medicine Greifswald, Greifswald, Germany; 6 Department of Health, Nursing and Administration, University of Applied Sciences Neubrandenburg, Neubrandenburg, Germany; 7 Department of Functional Genomics, Interfaculty Institute for Genetics and Functional Genomics, University Medicine Greifswald, Greifswald, Germany; 8 Integrated Research Biobank, University Medicine Greifswald, Greifswald, Germany

**Keywords:** Maternal mental health, Pregnancy, Intervention, Ecological momentary assessment, Attachment, Cortisol, Oxytocin, Human milk, Infant development, Longitudinal

## Abstract

**Background:**

As early as pregnancy, maternal mental stress impinges on the child’s development and health. Thus, this may cause enhanced risk for premature birth, lowered fetal growth, and lower fetal birth weight as well as enhanced levels of the stress hormone cortisol and lowered levels of the bonding hormone oxytocin. Maternal stress further reduces maternal sensitivity for the child’s needs which impairs the mother-child-interaction and bonding. Therefore, prevention and intervention studies on mental stress are necessary, beginning prenatally and applying rigorous research methodology, such as randomized controlled trials, to ensure high validity.

**Methods:**

A randomized controlled trial is used to assess the impact of psychotherapy and telemedicine on maternal mental stress and the child’s mental and physical health. Mentally stressed pregnant women are randomized to an intervention (IG) and a not intervened control group. The IG receives an individualized psychotherapy starting prenatal and lasting for 10 months. Afterwards, a second randomization is used to investigate whether the use of telemedicine can stabilize the therapeutic effects. Using ecological momentary assessments and video recordings, the transfer into daily life, maternal sensitivity and mother-child-bonding are assessed. Psycho-biologically, the synchronicity of cortisol and oxytocin levels between mother and child are assessed as well as the peptidome of the colostrum and breast milk, which are assumed to be essential for the adaptation to the extra-uterine environment. All assessments are compared to an additional control group of healthy women. Finally, the results of the study will lead to the development of a qualification measure for health professionals to detect mental stress, to treat it with low-level interventions and to refer those women with high stress levels to mental health professionals.

**Discussion:**

The study aims to prevent the transgenerational transfer of psychiatric and somatic disorders from the mother to her child. The effects of the psychotherapy will be stabilized through telemedicine and long-term impacts on the child’s and mothers’ mental health are enhanced. The combination of psychotherapy, telemedicine and methodologies of ecological momentary assessment, video recording and bio banking are new in content-related and methodological manner.

**Trial registration:**

German Clinical Trials Register: DRKS00017065. Registered 02 May 2019. World Health Organization, Universal Trial Number: U1111–1230-9826. Registered 01 April 2019.

## Background

Perinatal stress and mental strain can impact negatively on the mother’s and the offspring’s mental as well as physical health. Hormonal changes, critical life events and socio-economic disadvantages like unemployment, poverty and discrimination increase emotional distress and the risk of psychiatric disorders during pregnancy [1–4].

The WHO reports an estimated 10% of mental disorders worldwide during pregnancy, 15.6% in developing countries. The percentages are even higher after childbirth [5]. Reimbursement data of a German statutory health insurance demonstrates that 43.6% of pregnant women received at least one psychiatric diagnosis. Antenatal depression affects 9.3% of women, anxiety 16.9%, somatoform/dissociative disorders 24.2%, and acute stress reactions 11.7% [6]. These data concur with those of international studies reporting prevalence rates for antenatal depression of 11% and for anxiety disorders of 15% [7]. Moreover, pregnancy itself seems to be a sensitive time for the development of mental disorders, as the prevalence of depression is higher in the third (12.0%) and in the second (12.8%) than in the first trimester (7.4%) [8]. Women suffering from mental disorders also display poorer physical health, with higher risk of preeclampsia, reduced sleep, increased substance use, elevated cortisol levels and below-average weight gains [9–12].

In addition to the burden on pregnant women, maternal stress and mental disorders during pregnancy also affect birth outcomes as well as the physical health and the cognitive development of the offspring via intergenerational transmission processes. This transgenerational transmission occurs via placental blood exchange, fetal adaptation processes, epigenetic and hormonal programming, circumstances of the birth and impaired mother-offspring interactions [13–15]. It is supposed that elevated cortisol levels in stressed pregnant women may cause placental hypofusion and increased fetal circulation of glucocorticoid which impact on fetal development [3, 15]. This raises the risk of fetal growth restriction, preterm births and lower fetal birth weight [16–20]. Low birth weight neonates therefore exhibit an increased risk for diagnosis of infectious diseases, strokes, obesity, cancers, mental and behavioral disorders [21, 22].

Moreover, due to the maternal mental stress, the sensitivity for the emotional and developmental needs of the offspring is reduced which impairs mother-child-interaction and the bonding process [23–26]. Consequently, the social, psychological and cognitive child development is impaired associated with an increased risk for the child to develop own psychiatric and somatic disorders [27–32]. Recent studies examining this transgenerational transmission of disorders report that infants of mothers with antenatal and prenatal psychiatric disorders are at higher risk for regulatory disorders, ADHD, conduct disorders, elevated cortisol reactions to stress as well as impaired attachment styles and heightened hypochondriacal symptoms [12, 33–41].

### Prevention and intervention of prenatal and postpartum mental stress

These interdependencies raise the question how maternal mental health can be improved and how maternal distress and mental disorders can be prevented. A Cochrane Review provides evidence that psychotherapeutic and psychosocial interventions for postpartum depression lead to a reduction of depressive symptomatology of 30% [42]. Especially cognitive-behavioral therapy (CBT), individual treatments and a start of interventions in late pregnancy are effective for treating and preventing depressive symptoms perinatal and postpartum [43–46]. Additionally, CBT is effective in clinical settings as well as community samples, in women with severe as well as mild depressive symptoms [45].

Separate components of cognitive therapy have been proven as effective as long-term CBT: women with preterm infants and financial hardship reported fewer depressive episodes and symptoms after problem-solving education [47]. Another prenatal intervention study implemented a depression, anxiety and parenting preparation program. Women received a community networking booklet and a self-help support workbook with regular individual telemedical support. The workbook was constructed to correspond to the telephone calls, for example, regarding instructions about problem-solving skills, the strengthening of protective factors and the reduction of risk factors. Women of the intervention group scored lower in levels of depression, anxiety, stress and parenting dysfunction as women in the routine care group [48]. In addition, there are several short-term interventions based on cognitive-behavioral therapy proven to be effective, for example interpersonal psychotherapy [46, 49] and mindfulness-based cognitive therapy [50, 51].

Research around fetal programming recommends intervention starts as early as possible, that means on preconception or in pregnancy [15, 52].

It can be summarized that pregnancy and the postpartum period are stages of life with increased risk to develop mental disorders. Prenatal und postpartum intervention programs have effects on mental health in reducing stress and depressive symptoms [53]. But there are manifold barriers for women to access treatments [54–56].

### Barriers to treatment seeking among pregnant women

Primarily, prevalence rates of mental disorders are high, but detection rates are generally low [57]. Thus, midwifes and obstetricians need to be trained to detect mental health problems and to offer support [52]. Socio-economically disadvantaged women living in rural areas are twice as likely affected by mental disorders, but for these women in particular it is difficult to engage and retain treatments [49, 58]. Time constraints, access to transportation, need for childcare, financial pressure are just some of those obstacles. Short-term interventions with the possibility of home visits are therefore prone to be more effective in this population [49, 52]. Telemedical interventions are welcome opportunities to support mentally stressed women on-site in their every-day life [48, 59–61]. Thus, a combination of cognitive behavioral therapy and telemedical follow-up intervention seems to be promising in treating maternal mental stress.

### Research agenda of the current study (PriVileG-M trial)

The study PriVileG-M focuses on a population of women living in a rural area (Western Pomerania in Northern Germany) with high rates of unemployment and poverty [62]. Mentally stressed women are detected during pregnancy by short screening questionnaires in midwife or obstetrician surgeries during standard medical examinations, and receive a psychotherapeutic CBT short-intervention with subsequent telemedical support. Thus, the study offers individualized visiting support rather than waiting for the concerned to ask for help.

Limits of previous studies exploring intervention effects on prenatal and postpartum mental health are small sample sizes, non-random designs, lack of control groups and an absence of follow-up data [63]. PriVileG-M is therefore conceptualized as a sequentially randomized controlled trial with a randomized control group of mentally stressed women receiving no intervention and an additional, non-randomized control group of mentally healthy pregnant women. The intervention group receives a psychotherapeutic treatment. Approximately 10 months after baseline, the intervention group is randomized again in two groups with and without telemedical intervention. Women are monitored through prenatal and postpartum period by four follow-ups, the last occurring 1 year after birth. Following the interventional study, a qualification measure will be designed. Health professionals who regularly are in touch with pregnant women (e.g., midwives, nurses, social workers) are trained to detect mental disease symptoms, to relieve their psychiatric strain and to refer to mental health professionals if necessary. The aim is to detect mental stress and to offer treatment as early as possible. Thus, mental stress in mothers and children might be reduced and long-term effects prevented. During the qualification measure, basic knowledge about psychiatric diseases, existing supply structures and therapy options are taught. Therefore, specific themes raised during the intervention study will be extracted and discussed. Further, short, easy to learn and customizable interventions will be taught, for instance, emotional and stress regulation, social skills training or relaxation techniques.

### Objectives

The present study examines the impact of individualized psychotherapy and telemedicine on prenatal and postnatal stress and mental health via a sequentially randomized controlled trial. The study aims to explore, and ideally prevent, transgenerational transfer of physical and mental stress and disorders from the mother to her child. Moreover, the results will be used to develop a teaching course for professionals that work with pregnant women and young mothers (e.g., social workers, midwives, nurses) to help them in recognizing symptoms of mental illness and offering or referring to appropriate care.

#### Primary outcomes

(1) Concerning the women, interventions are expected

i. to reduce mental stress and to improve mental health and quality of life
ii. to enhance self-awareness, self-confidence, self-efficacy and resilience

(2) Concerning the children, interventions are expected

i. to reduce rates of preterm birth as well as complications during childbirth
ii. to reduce regulation disorders in children
iii. to reduce cognitive, physical and psychomotor retardations
iv. to reduce infectious diseases and low fetal birth weight

(3) Concerning women and children, interventions are expected

i. to improve mother-child-interaction and to strengthen bonding
ii. to lower cortisol levels in both mother and child

The outcomes (1) to (3) are expected in the groups of mentally stressed mothers to converge to those of healthy mothers and children in the non-randomized control group at the end of the study. In contrast, it is assumed that these outcomes differ from the control group of stressed women without intervention from those of the intervention group.

(4) In addition, the results of the study will lead to the development of a qualification measure for health professionals who will be trained to detect mental stress, and in turn to refer those women to mental health professionals.

#### Secondary outcomes

The study also aims to examine hormonal transmissions between mother and child to explore neurobiological mechanisms of transgenerational transmission. Therefore, a biobank will be built up to analyze DNA-sequences, RNA-expression, miRNA as well as DNA methylation and other metabolomics. In particular, the study scrutinizes how the development of the HPA-axis in infants is shaped by depressive symptoms of the mother and how the HPA-axis in mother and infants is influenced by the different interventions of the study. Moreover, in exploratory analyses, the constituents of breast milk will be analyzed. It is hypothesized that oxytocin levels will increase and melatonin levels will decrease in both stressed mothers and child after treatments. Finally, it is assumed that the intervention increases rates of breastfeeding mothers in the intervention group.

## Methods/design

Pregnant women are screened in midwife or obstetrician surgeries. On the basis of the screening results, the women are assigned to the group of healthy or to the group of mentally stressed women. While the healthy group serves as an additional control group (HCG) over the course of the study, the group of mentally stressed women will be randomized in two steps. In a first step of the randomization process, mentally stressed women are randomized to a psychotherapeutic intervention group (PIG) or a psychotherapeutic control group (PCG) with usual care. In a second step following the psychotherapeutic intervention, women of the PIG are randomized to a subsequent telemedical intervention (TIG) or a telemedical control group (TCG). All participants will be assessed at five time points: baseline (prenatal), around the delivery, 8 weeks, 26 weeks and 52 weeks postpartum (see Table 1). The SPIRIT statement for clinical trial protocols is used to report methods and procedure of this study.

**Table 1:** Questionnaires and items employed at the different study assessments

Study Assessments	Measures	Study time points						
		S1	S2	BL^a^	0W	8W	26W	52 W
Socio-demographic	Date of birth of the mother	X						
	Date of birth of the infant	X						
	Week of gestation	X	X	X				
	Educational attainment	X		X				
	Marital status	X		X				
	Indigenous status			X				
	Profession			X				
Medical								
Physical health of the mother	Medical documentation of the mother from the German Mutterpass				X			
	Alcohol, Smoking and other Lifestyle habits, chronic diseases, pregnancy related diseases			X	X	X	X	X
	Documentation of medication using Brown bag review			X				
Physical health of the infant	Child’s medical records, the Geman U-Untersuchungs-Heft filled in by pediatrician					X	X	X
	Semi structured health interview including number of infectious diseases, chronic disease use of antibiotics.					X	X	X
	Brown bag review for medication					X	X	X
	Anthropometric measures including length, weight, and head circumference					X	X	X
	physical examination of the infant					X	X	
Infant General Development	Neuropsychologic Development Screening (NES)					X	X	X
Infant behavior	German questionnaire about crying, feeding and sleeping behavior (SFS)					X	X	
	Infant Behavior Questionnaire (IBQ-R)					X	X	X
Breastfeeding Rate	Questionnaire about breastfeeding rates and times					X	X	X
Psychological								
Stress Symptoms	Brief Symptom Inventory (BSI-18)	X	X	X		X	X	X
	Coping Inventory for Stressful Situations (CISS)			X				X
	questionnaire about chronic stress (TICS)			X				X
	Recognition of stress-factors (KFB)			X		X	X	X
Resilience	RS-11			X		X	X	X
Mental Health								
Mental Disorders	Diagnostic Interview for Mental Disorders (DIPS)		X					
Trauma	Childhood Trauma Questionnaire (CTQ)			X				
Dissociation	Dissociative Experiences Scale-Taxon (DES-T)			X			X	X
Depression	Patient Health Questionnaire (PHQ-9)			X		X	X	X
	Edinburgh Postnatal Depression Scale (EPDS)			X	X	X	X	X
Worries	Penn State Worry Questionnaire (PSWQ)			X		X	X	X
	Cambridge Worry Scale (CWS)			X				
Personality Traits	NEO-FFI			X				X
Social network	short partnership questionnaire (PFB-K)			X				
	Perceived Social Support Questionnaire (F-SozU-6)			X		X	X	X
Attachment in adults	Experiences in Close Relationships - Revised (ECR−RD 12)			X				
	Revised Adult Attachment Scale (AAS-R)			X				
Prenatal attachment	Maternal Antenatal Attachment Scale (MAAS)			X				
Postpartum attachment	Postpartum Bonding Questionnaire (PBQ)					X	X	X
Video assessments								
Attachment	Mother-child interaction					X	X	
Ecological Momentary Assessment								
Mental state	Multidimensional Mood Questionnaire (MDMQ)			X			X	X
	stress level			X			X	X
Physical health	self-related health of the mother			X			X	X
	Of the infant						X	X
Mother-child-interaction	Modified items of the PBQ						X	X
	Modified item of the self-assessment questionnaire about mother-child-interaction (SF-MKI)						X	X
Mother-child-bonding	Modified items of the MAAS			X			X	X
Child sleeping behavior	Modified items of the regulation disorders questionnaire (SFS)						X	X
Laboratory								
Saliva Cortisol Mother and Infant	At midday collection during visit			X		X	X	
	Morning and evening Saliva cortisol from 3 different days					X	X	
Saliva Melatonin	Saliva melatonin at midday during the visit			X		X	X	
	Morning and evening Saliva Melatonin from 3 different days					X	X	
Urin sample Mother and Infant	Analysis of metabolics and oxytocin			X		X	X	
Bloodsample of the mother	Full blood analysis including complete blood count, electrolytes, glucose, kidney function, liver, thyroid and inflammatory values			X			X	
	Mothers Epigenetics			X			X	
Cordblood	Infants Epigenetics				X			
Colostrum	Composition of Colostrum, Peptidom				X			
Breast milk	Composition of Mothermilk, Peptidom					X	X	
Evaluative Assessments								
Qualitative interviews	Structured individual interviews about the contentment of the treatment result, perceived benefits and changes					X	X	X
Quantitative questionnaires	expectations in the study			X				X
	perceived benefits of the study and the quality of the interventions					X	X	X
	impact of the telemedical intervention on quality of life							X
	study evaluation							X
Documentation								
Therapeutic process^a^	date, time, home visit or not, woman appeared or not, treatment focus, course of content, changes since the last consultation, agreements and homeworks, interactions, hypotheses, discussed topics, therapeutical interventions							
Telemedical process^a^	date, time of day, duration of the call, circumstances of the call							

Note: S1 = Screening1, S2 = Screening2, BL = Baseline (occurs at approximately 25-28 weeks gestation), 0W = delivery, 8W = 8-weeks-follow-up, 26W = 26-weeks-follow-up, 52W = 52-weeks-follow-up, ^a^documentation of the treatment process (therapeutical and telemedical) do not occur at follow-ups, but over the period of the treatment at each consultation

### Study participants

#### Power and sample size

**Fig. 1:**
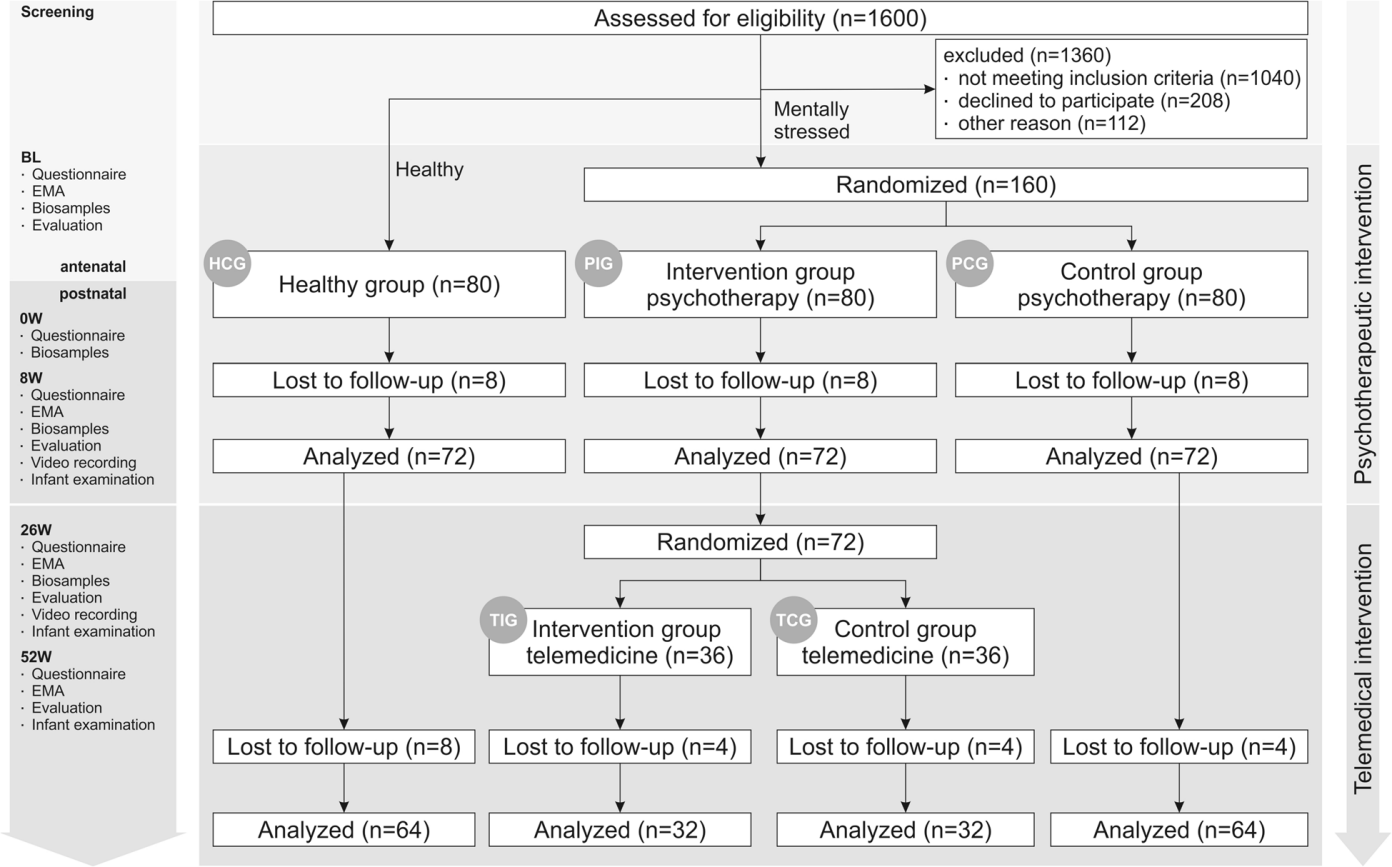
Study procedure, assessments, interventions and flow diagram of the randomized controlled trial. Note: BL = Baseline (occurs at approximately 25–28 weeks gestation), DL = delivery, 8 W = 8-weeks-follow-up, 26 W = 26-weeks-follow-up, 52 W = 52-weeks-follow-up, n = number

The primary outcome criterion of the study is to reduce mental stress of the women assessed by the brief symptom inventory (BSI-18). Former research reported healthy women to have a BSI-18 total score of 3–4 points, whereas mentally stressed women had 15–25 points [64]. After psychotherapy the BSI-18 total score was reduced by 10 points [65, 66]. Hence, assuming a medium effect size (Cohen’s d = 0.5 corresponding to a reduction by 7 points), a test-retest correlation of 0.5 between the baseline assessment and 26-week-follow-up, a significance level α = 0.05 and a power of β = 0.80, 27 women are required in each group. As telemedicine is an add-on focusing on stabilizing the effects of the psychotherapy, the second randomization into TIG and TCG is neglected in this calculation. However, envisaging 80 women for each group and assuming a drop-out-rate of 10%, 36 women are randomized to the TIG and TCG which is enough to analyze medium to large effects. Figure 1 shows the participant flow and the sample sizes of the different study groups.

#### Inclusions criteria

Pregnant women are included if they are between 17 and 45 years of age and at the end of the second trimester (25th to 29th week of pregnancy). For the psychotherapeutic intervention group (PIG) and the psychotherapeutic control group (PCG), mental stress is the primary inclusion criterion (BSI-18 total score ≥ 7 or BSI-18 sub scales ≥3 and/or psychiatric diagnosis according to the diagnostic interview (DSM-IV/ICD-10) [67]. In the control group of healthy women, women may not report elevated mental stress, neither in the screening questionnaire (BSI-18 total score < 7) nor in the clinical interview.

#### Exclusion criteria

Women with multiple pregnancies or foreseeable major birth defect of the fetus, as well as women experiencing symptoms of withdrawal, exhibiting low motivation for therapy, suffering alcohol or drug addiction, having severe psychosis or reporting suicidality will be excluded. In addition, women being submitted to a recent psychiatric or psychotherapeutic treatment (or another study including such treatments) have to be excluded in order to impede confounding of therapeutic effects. However, if women initiate psychotherapeutic treatment during the study, they will not be excluded from the study.

### Recruitment

According to the statistical office of the federal state of Mecklenburg-Western Pomerania, about 13,000 children are born in Mecklenburg-Western Pomerania per year thereof 25–30% in the study region Western Pomerania [62]. In Germany as a whole, the 12-month prevalence of psychiatric disorders was estimated at 30–35% including a 12-month prevalence of addictive disorders estimated at 5–10% [68]. Hence, we expect 20–30% of the pregnant women to have a mental illness, resulting in approximately 650 childbirths of mentally stressed women in Western Pomerania per year. It is aimed to screen 1600 pregnant women.

The screening procedure is staggered in two phases: i) Pregnant women at the beginning of their second trimester consulting gynecologists, obstetrics centers, hospitals and/or counseling centers receive information material and the primal screening questionnaire on site to assess the first set of in- and exclusion criteria (multiple pregnancies, age, pregnancy trimester, BSI-18 total score). This primal screening questionnaire is evaluated by the study staff. ii) Women who gave their written consent to be contacted and are eligible to the study are invited for a detailed assessment of the eligibility criteria at the end of their second trimester of pregnancy. Women meeting the inclusion criteria accomplish the baseline assessment afterwards. On this level, mentally stressed (BSI ≥ 7 and/or psychiatric diagnosis) and healthy pregnant women are recruited with a 2:1 ratio.

### Allocation

After the baseline assessment, mentally stressed women are randomly allocated to the psychotherapeutic intervention group (PIG) or the psychotherapeutic control group (PCG) with a 1:1 allocation ratio. Healthy pregnant women are assigned to the healthy control group (HCG).

Six months postpartum, women of the PIG are again randomized and receive either a subsequent telemedical intervention of 6 months duration (TIG) or not (TCG) with an allocation ratio of 1:1. An independent researcher who is not involved in the PriVileG-M trial prepares the allocation by the statistical software SAS Version 9.4 (TS Level 1 M3 Copyright© 2002–2012 by SAS Institute Inc., Cary, NC, USA) using a randomization list with simple random sampling.

### Blinding

Study information to participants includes information about the respective study arms and the randomized design. An independent researcher is responsible for the allocation by the SAS computer program. Participants, psychotherapists and telemedicine nurses are not blinded for the group allocation, but data collectors are not informed about group allocation.

### Interventions

#### Consultation

After study inclusion, all participants receive a semi-standardized consultation from a study psychologist. Medical and psychological information on pregnancy, childbirth, and puerperium are provided. The impact of one’s own health behavior on the (unborn) child is explained and discussed. Finally, the women receive advise about existing supply structures and assistance beyond the study (e.g. counseling centers, psychiatric out- and inpatient clinics, family assistance). Contact data of a consultant who can be contacted in crises situations are provided both verbally and in writing.

#### First level allocation: psychotherapeutic intervention

Women of the PIG are allocated to an individualized psychotherapeutic intervention based on the principles of cognitive-behavioral therapy (3–4 months prenatal) which lasts for up to 10 months (5–6 months postnatal). Utilization will be tracked to assess implementation adherence. Participants will receive up to 20 individual biweekly sessions of 50 min each. Although it is strived to implement most of the therapy meetings in the study center, home visits are possible if necessary due to the socio-economic or health conditions.

The aim of this psychotherapeutic intervention is to reduce mental stress and psychiatric symptoms and to enhance self-efficacy and self-confidence. The therapeutic intervention is based on behavioral and supportive elements, for instance, psychoeducation, social skills training, stress and emotion regulation. A high individualization of the therapy is planned to address the heterogeneity of psychiatric symptoms, socio-economic status and conflicts of the women. Similar to traditional psychotherapy, the content and process of the intervention are individually adjusted to the symptoms, needs, and progress of the women. Because the father of the child, the partner of the mother or other family members influence the well-being of the pregnant women, meetings with the couple or the family will be realized upon demand. In case of crises situations, the women have the opportunity to contact a clinical psychologist/psychotherapist and to demand a crisis meeting with their psychotherapist.

#### Subsequent allocation: telemedical intervention

After finishing the psychotherapy approximately 6 months after childbirth, women of the PIG are randomized either to the TIG or the TCG. Women of the TIG are regularly contacted by trained nurses via telephone call and additional short message to stabilize the effects of the psychotherapy and support the women in mastering daily life challenges [48, 59]. Whereas calls are made biweekly during the first 2 months and 4-weekly afterwards, short messages are sent weekly. The content of the telephone calls and short messages are leaned to the content of the psychotherapeutic intervention. The calls are semi-structured. During the first structured part, nurses assess the current mental health (BSI-18) as well as current positive and negative life events. In the second part, women have the possibility to discuss issues and projects in supportive dialogues.

The TCG is not receiving further interventions. The telemedical intervention lasts for 6 months and terminates the interventional part of the study.

### Assessments

All participants are investigated five times during the study from the end of the second pregnancy trimester (25th -29th week of pregnancy) until 1 year postpartum. The baseline assessment (BL) will take place between the 25th and the 29th week of pregnancy, the birth-follow-up at 24–72 h after delivery (0 W), the 8-week-follow-up at 8 weeks postpartum (8 W), the 26-weeks-follow up at 6 months postpartum (26 W) and the 52-weeks-follow-up 1 year postpartum (52 W). The baseline assessment, the 26-week- and 52- week-follow-up are followed each by an Ecological Momentary Assessment (EMA) lasting 10 days.

At the assessment points mental health, physical health and interaction of mother and infant are investigated using standardized interviews, questionnaires, video recordings and the extraction of biosamples. Additionally, process evaluation questionnaires and interviews are conducted. The development and health of the child is evaluated in a physical examination.

For an overview of the assessment time points and assessments made, see Fig. 1.

### Outcome measures

Table 1 shows the maternal and infant variables investigated at different time points during the study. They can be classified into medical, psychological, video, ecological momentary, laboratory and evaluation assessments as well as the documentation process of the treatments.

#### Medical assessments

Pregnancy complications, course of the delivery, and infant physical health are assessed using medical documentations of the mother (maternity records and the obstetric clinical records the so called “Mutterpass”) and the infant (child’s medical records, i.e. “U-Untersuchungsheft”). The „Mutterpass” is a medical record book for all German pregnant women that is completed by the gynecologist, the midwife, and the staff of the obstetric clinics [69]. The “U-Untersuchungsheft” includes information about the infant’s health and development that are assessed by the staff of the obstetric clinics and by pediatricians at routine pediatric examinations until the age of six [70].

Furthermore maternal health, infant health and feeding patterns are assessed through a structured maternal interview at each assessment point to obtain medical details. During an additional physical examination by a pediatrician at 8 W and 26 W neurological development, anthropometric measurement and physical examination focusing on skin, respiratory tract, abdomen and circulatory system will take place.

The children’s developmental levels, temperament and possible early regulatory problems are evaluated by a German Development Screening (NES) [71], a German questionnaire assessing problems of excessive crying along with sleeping and feeding difficulties (SFS) [72] and the German version of the Very Short Form of the Infant Behavior Questionnaire-Revised (IBQ-R) [73].

#### Psychological assessments

An overview of all psychological assessments is given in Table 1. Stress symptoms are assessed by different questionnaires: the BSI-18 [64, 74], a questionnaire about chronic stress (TICS) [75], a short questionnaire for recognition of stress-factors (KFB) [76] and the Coping Inventory for Stressful Situations (CISS) [77]. The RS-11 questionnaire is used to assess resilience as personality trait [78].

For the psychiatric diagnosis at the screening and baseline assessments, the Diagnostic Interview for Mental Disorders (DIPS) is used [67, 79]. The DIPS is a standardized interview assessing psychiatric disorders according to ICD-10 and DSM-V. In addition, symptoms of several mental disorders are assessed in detail: the Childhood Trauma Questionnaire (CTQ) is used to evaluate six early traumatic experiences and assesses individual’s understanding of their childhood trauma [80]. The Dissociative Experiences Scale-Taxon (DES-T) is applied for the assessment of pathological dissociative phenomena [81]. Depression symptoms are monitored by the Patient Health Questionnaire (PHQ-9) [82] and the Edinburgh Postnatal Depression Scale (EPDS) [83]. Worries are assessed with the ultra-brief version of the Penn State Worry Questionnaire (PSWQ) which evaluates pathological worries by 3 items [84]; and with the Cambridge Worry Scale (CWS) containing items about worries during pregnancy [85].

The NEO-FFI is a multidimensional personality inventory assessing individual differences in five dimensions, namely neuroticism, extraversion, conscientiousness, openness to experience and agreeableness [86]. Concerning social support and partnership status, a short partnership questionnaire (PFB-K) [87] and a brief form of the Perceived Social Support Questionnaire are used (F-SozU-6) [88].

There are two questionnaires assessing attachment in the adult women: the brief form of the Experiences in Close Relationships - Revised (ECR − RD 12) [89] and the Adult Attachment Scale in its revised version (AAS-R) [90, 91]. Mother-child bonding is assessed prenatal as well as postpartum. The Maternal Antenatal Attachment Scale (MAAS) evaluates the attachment of the mother with her unborn child [92]. The Postpartum Bonding Questionnaire (PBQ) is used to assess mother-infant bonding after childbirth [93].

Quality of life in general is monitored by the WHOQol-Bref [94]. The Mother-Generated Index (MGI) is used to assess qualitative as well as quantitative quality of life from pregnancy to the postpartum period [95]. The self-rated health status items examine the subjective perceived physical health of the women, in relation to the physical health of other pregnant women or young mothers [96]. Health Literacy is assessed by the HLS-EU-Q16 [97].

#### Video assessments

Maternal sensitivity for the child’s needs and the mother-child-interaction are assessed using video recordings under non-threatening conditions. The mother and child dyads are filmed for 3–5 min while playing or caring. Afterwards, according to standardized criteria, trained raters code the recorded interaction situations.

#### Ecological momentary assessments

Measurements used during the EMA are presented in Table 1. To assess the relation of mental stress, mother-child-interaction and mother-child-bonding during daily life as well as to assess the transfer of the interventional treatment into the daily life, after BL, 26 W and 52 W assessments, an EMA is implemented. Ten days with three daily time points (9:00, 14:00, 19:00) the women evaluate their mental state via the Multidimensional Mood Questionnaire (MDMQ) [98], stress level [99], self-related health [96] as well as bonding to their children with modified items of the MAAS. At time points 26 W and 52 W, additional questions are asked related to the child: mother-child-interaction via modified items of the PBQ and a mother-child-interaction questionnaire [93, 100], health [96] and sleeping behavior by modified items of the SFS [72].

#### Laboratory assessments

To analyze the impact of stress on stress sensitive physiologic systems, biosamples are taken from the mother and child. Additional we aim to set up a biobank for future researcher. For an overview see Table 1.

Saliva samples are taken at the BL from the mother and postpartum at 8 W and 26 W from mother and child to analyze for melatonin and cortisol. For saliva collection a small cotton swab remains in the mouth for 2 min.

To mitigate the influence of diurnal variations, all assessments take place in the afternoon between 13:00–16:00. Three saliva samples are taken during the assessment to evaluate the stress response to the mother-infant-interaction video recordings. Samples are directly stored on − 80 °C until further analysis.

Circadian rhythmic of cortisol and melatonin is studied in saliva samples from mother and child taken at home 30 min after waking and 30 min before bedtime on 3 days the week after the 8 W and 26 W assessment. These samples are stored in the refrigerator until the day of assessment and then stored on – 80 °C until further analysis.

Blood samples are taken from the mother at BL and 26 W. First, a direct blood test of the complete blood count, electrolytes, glucose, kidney function, liver, thyroid and inflammatory values will take place. Second blood samples are stored for later epigenetic analysis.

Directly after cord clamping, cord blood is collected by the midwife, prepared for DNA analysis and stored in the biobank.

Postpartum colostrum and breastmilk for peptidome analysis are collected 24–72 h after delivery and if the infants are still breastfed at 8 W and 26 W postpartum. If there is breastmilk left over up to 10 ml are stored in our biobank.

Urine samples are collected and stored at BL, 8 W and 26 W from mother and child for future analysis of metabolic products and oxytocin [101].

#### Process evaluation

The evaluation of the study interventions will be implemented by mixed methods with quantitative analysis of study data and qualitative interviews. Ten women of the intervention control groups (PCG and TCG) and 10 of the intervention groups (PIG and TIG) will be randomly selected to participate in structured individual interviews at weeks 8, 26 and 52 postpartum. The interviews will last for 1 h and contain questions about the treatment as well as perceived benefits and changes.

The women of the PIG, TIG and the PCG and TCG will complete four quantitative evaluation questionnaires at different time points: (1) about the expectations in the study (BL and 52 W), (2) about the perceived benefits of the study and the quality of the interventions (8 W, 26 W, 52 W), (3) about the impact of the telemedical intervention on quality of life (52 W) and finally (4) a study evaluation questionnaire at the end of the study (52 W).

#### Documentation of the psychotherapeutic treatment process

The following process data are assessed at each psychotherapeutic consultation: date, time, home visit or not, woman appeared or not, treatment focus, course of content, changes since the last consultation, agreements and homeworks, interactions, hypotheses, discussed topics, therapeutical interventions.

#### Documentation of the telemedical treatment process

For each telephone contact, process information like date, time of day, duration of the call, circumstances of the call (was it scheduled or unscheduled, was the caller the nurse or the participant, which nurse conducted the call) will be documented with the computer-aided documentation system. The scheduled intervention calls start openly, starting with questions about everyday life (e.g. “How do you do feel today?”, “What went well today or yesterday?”, “Are there any problems?”). Within the psychotherapeutic intervention group, therapeutic goals will be identified and transferred to the telemedical intervention. These therapeutic goals will be addressed during the regularly telephone calls. For each of these therapeutic goals the participants shall summarize how well they performed on the specific therapeutic goal and evaluate their efforts on the goals on a scale from 0 (very well) to 4 (inadequate). It also is possible to include new goals if new problems or topics occur. The nurses will have the possibility to consult the psychotherapists if necessary. This open conversation is followed by the assessment of the women’s mental health by the BSI-18 [64, 74].

The intervention call ends with some concluding remarks (e.g. “What are you taking with you from the call today?”, “Would you like to discuss another topic next time?”, “Would you like to ask me something or do you have another wish yet?”). Afterwards, an appointment for the next call is made.

The weekly text messages take up global themes (e.g. “I wish a good start in the week.” Or individual messages such as “You wanted to go to a toddlers’ group. Did you go and how was it?”). The participants can answer and the nurses will respond if necessary or appropriate. Every contact via text message will be documented in the computer-aided documentation system.

### Data management

Data of all assessments and of the study interventions are documented using electronic case report forms. Plausibility check is carried out as soon as the data are entered into the system. Data are stored in a central study database based on the data management system of the Institute for Community Medicine of the University Medicine Greifswald according to current standards of data security laws. The research data analyzing happens only pseudonymized, which means separating identifying data from medical data.

All study information on paper, including written informed consent, screening surveys and questionnaires will be stored in locked file cabinets in areas with limited access.

Biosamples are stored under a pseudonym in refrigerators at the Institute of Clinical Chemistry and Laboratory Medicine of the University Medicine Greifswald with limited access.

Videos are recorded with a camera. Video data and those of the EMA are directly saved and encrypted within the study data management.

Women included in the study give written informed consent following comprehensive oral and written information about the study. For the participation of the child, all legal guardians give written informed consent. If a woman limits the given consent, the restriction is documented and further data assessment is stopped according to the restrictions made. If a woman withdraws the given consent at any stages of the study, all data are removed, excluding data published before the withdraw. Data are exclusively analyzed if pseudonymized. Publications use aggregated data not allowing personal identification. If data is shared it will only be shared without participant’s identifiers. Competent data management and the documentation process is supervised by a medical documentation specialist.

### Data monitoring & collection

A data monitoring committee (DMC) is not installed, as there is no blinding and adverse events can be directly reported to study employees. All aspects of the intervention fall within the scope of conventional psychiatric and maternity care. No interim analyses are planned, and all outcomes will be analyzed following data collection.

### Auditing

The trial audits will be conducted quarterly by the funding. The investigators report the current study status, the progress of each project partner and the publication progress.

### Harms

To meet acute situation of severe mental stress a telephone hotline is established. Moreover, the psychotherapeutic intervention is limited to 20 sessions. Thus, severe mental symptoms cannot be treated in this short period of time. But starting nevertheless the treatment of such symptoms or severe interpersonal conflicts entails the risk of iatrogenic effects, like worsening of symptoms. Further in collaboration with the psychiatric outpatient clinic of the University Medicine Greifswald, a counseling in the near term is possible for all participants in self endangerment.

To avoid harm from medical assessment, physical examination and venipuncture are only performed by trained health professionals.

#### Statistical methods

To answer the various research questions, the following methods will be used: regression analyses, multivariate analysis of variance with repeated measurements, correlation analysis, multilevel analysis and Chi-square tests. The results of the qualitative evaluation interviews will be analyzed by qualitative content analysis according to Mayring [102].

## Discussion

Primary aim of the study is to improve the mental health of the mothers and to generate valid data investigating the relation between maternal mental, physical and biological health and early psychobiological outcomes of the child as well as the mother-child-interaction and the mother-child-bonding. Implementing the psychotherapeutic intervention already during pregnancy encourages the development of adaptive health behaviors and favorable development environments to ensure a good start in life and healthy development for the children. Further, as the study is seeking to improve the mental health, supportive and emotional behavior of the mother, the mother might become a positive model for her child. Hence, in the medium and long-term, the study would ensure the children’s wellbeing and reduce problem behaviors of the child. Thus, the study has the notation to prevent the transgenerational transfer of psychiatric and somatic disorders [43–46].

Using telemedicine, the effects of the psychotherapy are sustainable stabilized and thus positive long-term impacts on the child are fostered [48, 59]. Further, the evaluation of the transfer of the intervention effects into daily life is supported by the ecological momentary assessment as well as the video recording of the mother-child-interaction. The combination of these methodologies is complex, heretofore unseen methodological approach to this topic. Moreover, comparing the endocrine profile of mother and child and analyzing the peptidome of the colostrum and breast milk has the potential to deepen the understanding of transgenerational transmission of stress.

### Limitations and generalizability

Women of the control group are not treated within the study but are referred to professional assistance at the end of the study. Further, it is unknown yet how healthy women will react to childbirth and study procedures (regarding mental stress and endocrine changes). Hence, differences between groups might be diminished. All women are recruited in Western Pomerania, a more rural area of Germany with more socio-economic disadvantages and a higher prevalence of psychiatric and somatic diseases [62]. Thus, the study sample might be affected more significantly which would make the conclusions on mildly affected women more difficult.

In addition, the study sample is restricted in its representativeness by some of the exclusion criteria. Women with multiple pregnancies, major birth defects, alcohol or drug addiction, psychotic symptoms as well as suicidality are particularly in need of interventions, but excluded in this study. Further, teenage mothers are a special target group not considered in the present study. Due to the young age, teenage mothers often are overwhelmed by the new responsibilities and insufficiently progressed in their emotional and personality development [103].

Although the application of the psychotherapeutic intervention and study assessments is envisaged to take place in the study center at the University Medicine Greifswald, home visits will be offered. Not all women are recruited from urban regions and some might be unable to visit the study center due to socio-economic, medical or personal reasons [49, 52]. However, home visits are more time consuming and some of the study assessments (e.g., the immediate processing of blood sample to measure oxytocin levels) are not possible at home visits.

As the study is very comprehensive, the multiple assessments could elevate the maternal mental stress level which in turn can result in increased drop-out rates.

Although couple and family meetings are feasible during the individualized psychotherapy, the study interventions are focused on the mothers and not primary family based. Hence, the active role of the fathers might be understated. In addition, a recent study suggests that even maternal preconception mental health predicts infant outcomes [104]. Consequently, prevention and intervention programs might have to start earlier as in pregnancy, even in the preconception phase.

Nevertheless, the implemented interventions are near to usual psychotherapeutic and telemedical therapies and thus are generalizable to treatment options beyond the study. Moreover, the individualization of the interventions is in line with current efforts of individualized medicine.

In sum, the PriVileG-M study has the potential to examine rigorously intergenerational transmission of mental stress from the mother to her child, to revise effects of interventions and to conclude from these results contents for the development of a qualification measure.

## Data Availability

The study results will be released to the scientific public by conferences and international peer-reviewed impact journals as well as to the general public by presentations in health care organizations and national congresses. The study results will also contribute to the development of a qualification measure informing nurses, midwifes, pediatricians and mental healthcare practitioners about low-level interventions for stressed pregnant and postpartum women. The datasets generated during the current study are available from the corresponding author on reasonable request.

## Abbreviations

0 W: Birth-follow-up after delivery
26 W: 26-weeks-follow
52 W: 52-weeks-follow-up
8 W: 8-weeks-follow-up
AAS-R: Adult Attachment Scale - Revised
BL: Baseline assessment
BSI-18: Brief Symptom Inventory
CBT: Cognitive-Behavioral Therapy
CISS: Coping Inventory for Stressful Situations
CTQ: Childhood Trauma Questionnaire
CWS: Cambridge Worry Scale
DES-T: Dissociative Experiences Scale-Taxon
DIPS: Diagnostic Interview for Mental Disorders
DMC: Data Monitoring Committee
ECR − RD 12: Experiences in Close Relationships - Revised
EMA: Ecological Momentary Assessment
EPDS: Edinburgh Postnatal Depression Scale
F-SozU-6: Perceived Social Support Questionnaire
HCG: Healthy Control Group
IBQ-R: Infant Behavior Questionnaire-Revised
KFB: Questionnaire for recognition of stress-factors
MAAS: Maternal Antenatal Attachment Scale
MDMQ: Multidimensional Mood Questionnaire
MGI: Mother-Generated Index
NES: German Development Screening
PBQ: Postpartum Bonding Questionnaire
PCG: Psychotherapeutic Control Group
PFB-K: Short partnership questionnaire
PHQ-9: Patient Health Questionnaire
PIG: Psychotherapeutic Intervention Group
PSWQ: Penn State Worry Questionnaire
RS-11: Resilience Scale
SFS: Questionnaire for Crying, Sleeping and Feeding
TCG: Telemedical Control Group
TICS: Questionnaire about chronic stress
TIG: Telemedical Intervention Group
